# Water quality prediction in sea cucumber farming based on a GRU neural network optimized by an improved whale optimization algorithm

**DOI:** 10.7717/peerj-cs.1000

**Published:** 2022-05-31

**Authors:** Huanhai Yang, Shue Liu

**Affiliations:** 1 School of Computer Science and Technology, Shandong Technology and Business University, Yantai, Shandong, China; 2 Binzhou Medical University, Yantai, Shandong, China

**Keywords:** Variational modal decomposition, Gated recurrent unit, Water quality prediction, Whale algorithm, Wavelet packet denoising

## Abstract

Sea cucumber farming is an important part of China’s aquaculture industry, and sea cucumbers have higher requirements for aquaculture water quality. This article proposes a sea cucumber aquaculture water quality prediction model that uses an improved whale optimization algorithm to optimize the gated recurrent unit neural network(IWOA-GRU), which provides a reference for the water quality control in the sea cucumber growth environment. This model first applies variational mode decomposition (VMD) and the wavelet threshold joint denoising method to remove mixed noise in water quality time series. Then, by optimizing the convergence factor, the convergence speed and global optimization ability of the whale optimization algorithm are strengthened. Finally, the improved whale optimization algorithm is used to construct a GRU prediction model based on optimal network weights and thresholds to predict sea cucumber farming water quality. The model was trained and tested using three water quality indices (dissolved oxygen, temperature and salinity) of sea cucumber culture waters in Shandong Peninsula, China, and compared with prediction models such as support vector regression (SVR), random forest (RF), convolutional neural network (CNN), recurrent neural network (RNN), and long short-term memory neural network (LSTM). Experimental results show that the prediction accuracy and generalization performance of this model are better than those of the other compared models.

## Introduction

In the sea cucumber farming production and management process, water quality is an important factor affecting healthy sea cucumber growth. The most suitable water environment for sea cucumber farming requires pollution-free water quality, dissolved oxygen above 5 mg, water temperature 0–30 degrees (preferably 10–16 degrees), and salinity maintained above 25 parts per thousand. Therefore, accurate prediction of the development trend of water quality indicators such as dissolved oxygen, water temperature and salinity is of great significance for ensuring sea cucumbers growth in a suitable water environment. Water quality data are often affected by various natural environments, showing strong volatility and randomness in time series, making predictions more difficult. With the continuous improvement and development of artificial intelligence technologies such as deep learning, the accuracy of water quality prediction continues to increase. In recent years, many scholars have proposed many nonlinear prediction models based on artificial intelligence technology.  Noori, Kalin & Isik (2020) developed a hybrid water quality predictions model by combining a process-based watershed model and artificial neural network(ANN). Lu & Ma (2020) proposed two short-term water quality prediction models based on extreme gradient boosting (XGBoost) and random forest (RF). Bui et al. (2020) studied the application of four standalone and twelve hybrid intelligent algorithms in water quality prediction.  Aldhyani et al. (2020) studied the application of advanced artificial intelligence (AI) algorithms to predict the water quality index (WQI) and water quality classification (WQC).  Avila et al. (2018) studied the application of intelligent algorithms such as Bayesian networks and random forests in water quality prediction. Azimi, Azhdary Moghaddam & Hashemi Monfared (2019) studied the water quality prediction model using an artificial neural network and fuzzy clustering.  Shi et al. (2019) proposed a clustering-based softplus extreme learning machine(CSELM) method to predict the change trend of dissolved oxygen concentration in aquaculture.  Xu & Liu (2013) combined the wavelet transform with the BP neural network to build the water quality prediction model. Zou et al. (2020) proposed a water quality prediction method based on a bidirectional long short-term memory network. Yan et al. (2021) proposed water quality prediction based on 1-DRCNN and BiGRU hybrid neural network model.

Synthesizing the above analysis, a large number of prediction methods based on artificial intelligence have been proposed for water quality prediction. All these methods have improved the accuracy of water quality prediction to a certain extent. However, there are many uncertain factors in sea cucumber farming water, and water quality time series are highly noisy and unstable; therefore, using the primary water quality series directly to establish prediction models is subject to substantial errors (Zhang et al., 2017). To improve the prediction accuracy, an effective method is to decompose the input data according to different fluctuation scales, extract components that are relatively stable and have different characteristic information, and then perform data noise reduction processing on each component. Commonly used data decomposition algorithms include empirical mode decomposition (EMD) (Huang et al., 1998; Ren, Suganthan & Srikanth, 2015), extended EMD (EEMD) (Wu & Huang, 2009), complete EEMD with adaptive noise (CEEMDAN) (Yeh, Shieh & Huang, 2010), empirical wavelet transform(EWT) (Gilles, 2013), and variational mode decomposition (VMD) (Dragomiretskiy & Zosso, 2014).

Ahmed et al. (2019) proposed a water quality prediction model based on neuro-fuzzy inference system and wavelet denoising technique. Eze et al. (2021) used EEMD and LSTM to form a chlorophyll-a concentration prediction model. Fijani et al. (2019) implemented a water quality parameter monitoring model based on the two-layer decomposition method (CEEMDAN and VMD) and extreme learning machine. Ren et al. (2020) proposed dissolved oxygen prediction in recirculating aquaculture systems based on VMD and a deep belief network (DBN). Barzegar, Asghari Moghaddam & Adamowski (2016) proposed wavelet-artificial intelligence hybrid models for water quality prediction. Liu, Xu & Li (2016) proposed a water temperature prediction model using empirical mode decomposition with back-propagation neural networks. Huan, Cao & Qin (2018) proposed a dissolved oxygen (DO) prediction model based on ensemble empirical mode decomposition (EEMD) and least squares support vector machine (LSSVM). Fan et al. (2021) proposed a hybrid prediction model based on wavelet decomposition(WD) and LSTM. These studies showed that a denoising algorithm based on data decomposition is a useful tool for time series preprocessing.

The EMD decomposition algorithm is prone to end effect and mode mixing problems. EEMD and CEEMDAN suppress the mode mixing problem to a certain extent, but there are problems of excessive decomposition and noise residue. The EWT algorithm needs to set the wavelet basis function, the number of decomposition layers and the noise reduction threshold in advance, and human factors have a greater impact on the decomposition results. VMD is a completely nonrecursive variational mode decomposition model (Lei, Su & Hu, 2019). By setting the parameters reasonably, VMD can effectively suppress mode mixing and end effect problems. In addition, there is no need to set wavelet functions in advance, and it can perform signal processing adaptively. VMD has advantages in processing nonstationary signals and suppressing noise.The above decomposition and denoising methods have good denoising effects, but they also have some shortcomings. In recent years, an increasing number of studies have shown that hybrid denoising methods have better performance than single denoising algorithms (Cao et al., 2021; Nie, Wang & Zhao, 2018; Fu et al., 2020). To effectively decompose and denoise sea cucumber aquaculture water quality data, this article applied a hybrid algorithm combining variational mode decomposition (VMD) and wavelet threshold denoising (WTD) to realize the denoising processing of water quality data.

In the prediction model based on intelligent calculation, the recurrent neural network (RNN) achieves good performance in the prediction of time series sequences. Long short-term memory neural network (LSTM) improve the structure of recurrent neural network. LSTM is a special RNN that solves the problems of gradient disappearance and gradient explosion during long sequence training. The principle of gated recurrent unit (GRU) is similar to that of LSTM, which simplifies the gating structure, which simplifies the gating structure by combining the forget gate and the input gate into an “update gate”, has fewer parameters than LSTM, and can achieve functions equivalent to LSTM in some applications. GRU (Gated Recurrent Unit) combines the unit state and the hidden state. Since the structure of the GRU network is simpler than that of the LSTM, it requires fewer parameters to adjust, and the training speed is faster, and the prediction performance is roughly equivalent to that of the LSTM. Therefore, the GRU recurrent neural network is used in this article to construct the water quality prediction model.

Like most neural network models, the prediction accuracy and stability of the GRU model are affected by its hyperparameter settings. In order to better solve the parameter optimization problem of intelligent models, a large number of intelligent swarm optimization algorithms have been proposed in recent years, such as Particle Swarm Optimization (PSO) (Kennedy & Eberhart, 1995), Grey Wolf Optimizer (GWO) (Mirjalili, Mirjalili & Lewis, 2014), Sparrow Search Algorithm (SSA) (Xue & Shen, 2020) and so on. Whale Optimization Algorithm (WOA) (Mirjalili & Lewis, 2016), a new type of algorithm, builds a model based on the hunting behavior of whales. It has the advantages of simple optimization mechanism, few adjustable parameters, and effectively avoid local optimization. The Whale Optimization Algorithm has the problems of slow convergence speed and reduced global optimization ability in the later stage of iteration. Therefore, this article improves the Whale Optimization Algorithm to elevate its optimization performance, and it uses the IWOA (Improved Whale Optimization Algorithm) to optimize the parameters of the GRU model and improves the prediction performance through the reasonable parameter configuration of the GRU model.

In this article, GRU is used to construct a prediction model to predict and analyze the changing trends of dissolved oxygen, water temperature and salinity in sea cucumber aquaculture water. To improve the prediction accuracy of the GRU model, this article uses VMD-WTD to effectively reduce the noise of the water quality data, and selects an improved whale algorithm to optimize the parameters of the GRU prediction model. The research contributions of this article are summarized as follows: (1) Using relative entropy to optimize the VMD decomposition parameters, realizes the joint noise reduction in VMD decomposition and wavelet threshold, and reduces the nonstationarity of water quality data and the influence of noise on the prediction results. (2) By improving the calculation method of the nonlinear convergence factor of the whale algorithm, the position update method of the whale algorithm is optimized, the search accuracy and breadth are improved, and the optimization performance of the algorithm is improved. (3) The improved whale algorithm is used to optimize the parameters of the GRU recurrent neural network prediction model, the optimal model structure and parameters are determined, and its convergence speed and prediction accuracy are improved. The rest of the article is structured as follows. The related theories, including VMD, wavelet threshold denoising, whale algorithm and GRU, are introduced in ‘Materials and Method’. The proposed prediction model is presented and compared with those of other existing methods in ‘Simulation Experiment and Result Analysis’. The conclusions are presented in ‘Conclusions’.

## Materials and Method

### Variational mode decomposition

VMD (Dragomiretskiy & Zosso, 2014) is a nonrecursive adaptive decomposition processing method that decomposes the input signal into different numbers of intrinsic mode functions (IMFs) through continuous iteration. Each mode component has a certain bandwidth and center frequency.In the VMD decomposition process, the number of modes *k* of the given sequence can be customized, and the optimal center frequency and limited bandwidth of each mode can be adaptively matched in the subsequent search and decomposition process.

Variational mode decomposition finds *k* mode functions with the smallest sum of estimated bandwidths, and requires the sum of all mode functions to be the original signal. The resulting constrained variational problem is shown in Eq. (1) (Rehman & Aftab, 2019).

(1) \begin{eqnarray*}\min \nolimits ({w}_{k},{u}_{k}) \left\{ \sum _{k}{ \left\| {\partial }_{t} \left[ ({\delta }_{t}+ \frac{j}{\pi t} )\ast {u}_{k}(t) \right] {e}^{-j{w}_{k}^{}t} \right\| }_{2}^{2} \right\} \end{eqnarray*}

\begin{eqnarray*}s.t.\sum _{k}{u}_{k}=x(t). \end{eqnarray*}

In the above formula, *x*(*t*) is the original signal, *k* denotes the total number of IMFs, and {*u*_*k*_} = {*u*_1_, *u*_2,_⋯*u*_*k*_} are the *k* IMF components obtained after decomposition. {*w*_*k*_} = {*w*_1_, *w*_2,_⋯*w*_*k*_} represents the corresponding central frequency of the IMF component. ∂_*t*_ denotes the differential processing of t, ${ \left\| \bullet \right\| }_{2}$ indicates 2-norm processing, *δ*_*t*_ is the Dirac function, *j* is the imaginary unit, and ∗ is the convolution operation (Niu, Xu & Wang, 2020). To solve the optimal solution of the abovementioned variational problem, the augmented Lagrange function is introduced, as shown in the following Eq. (2).

(2) \begin{eqnarray*}L(\{ {u}_{k}\} ,\{ {w}_{k}\} ,\lambda )=\alpha \sum _{k}{ \left\| {\partial }_{t}[({\delta }_{t}+ \frac{j}{\pi t} )\ast {u}_{k}(t)]{e}^{-j{w}_{k}t} \right\| }_{2}^{2}\nonumber\\\displaystyle +{ \left\| x(t)-\sum _{K}{u}_{k}(t) \right\| }_{2}^{2}+ \left\langle \lambda (t),x(t)-\sum _{K}{u}_{k}(t) \right\rangle .\end{eqnarray*}

In the above formula, *α* is the quadratic penalty term, *λ* is the Lagrangian multiplication operator, and 〈∗〉 denotes the vector inner product.

Using the alternating direction multiplier algorithm (ADMM), {*u*_*ik*_}, {*w*_*k*_}, and *λ* are iteratively updated to find the above variational problem and obtain the saddle point of the Lagrange function. When the accuracy requirements are met, the iteration stops, and finally, k optimal decomposition modal functions are obtained. The complete decomposition process is detailed in reference (Dragomiretskiy & Zosso, 2014).

### Relative entropy

Relative entropy is the quantification of the degree of difference between two probabilities (Zhu et al., 2021). Relative entropy can measure the difference and closeness of two probability distributions, and can be used as the loss function of some optimization algorithms. The relative entropy between the probability density functions *p*(*x*) and *q*(*x*) of the discrete random variable *x* is defined as formula ((3)):

(3) \begin{eqnarray*}D(p{|}{|}q)=\sum _{x\in X}p(x)log \frac{p(x)}{q(x)} .\end{eqnarray*}

The Variational Mode Decomposition (VMD) algorithm needs to pre-set parameters such as the number of modes *k* and penalty factor *α*. Studies have shown that the combination of *K* and *α* values has a significant impact on the decomposition accuracy. Using relative entropy to select the best combination of VMD parameters [*k*, *α*] can effectively avoid insufficient or over decomposition, and achieve reasonable decomposition of the data signal.

The implementation steps are as follows:

Step 1: The water quality data is decomposed according to a decomposition algorithm such as Empirical Mode Decomposition (EMD) that does not need to preset such parameters as the number of decomposition modes, and the max value of the decomposition mode number *k* of the relevant sequence is determined.

Step 2: The initial value of the penalty factor *α* is set to 1000 according to experience and the mode number parameter *k* is from two to the max value determined in step one, and then Variational Mode Decomposition can be multiply performed on the signal. The relative entropy of the modes obtained by each decomposition will be calculated, and the *k* value corresponding to the minimum relative entropy is the best parameter.

Step 3: After the mode number parameter *k* is determined, the range of *α* is set as [1000, 2000] according to experience, and *α* is incremented by 50 within the value range to perform multiple VMD decompositions. The relative entropy of each decomposed mode can be calculated, and then the optimal value of the penalty factor *α* can be determined according to the smallest relative entropy.

Step 4: By using the VMD’s optimal parameter combination [*k*, *α*], the water quality signal will be re-decomposed to obtain a more reasonable decomposition sequence.

### Wavelet threshold denoising

The essence of wavelet threshold noise reduction is to decompose the signal containing noise, and separate the signal and noise into wavelet packet coefficients with different amplitudes. The coefficients with smaller amplitudes contain more noise. A suitable threshold is used to strip the noise and retain the useful signal, to realize the denoising processing of the original signal. In the process of wavelet threshold denoising, the threshold function choice is very important. Wavelet threshold processing methods are divided into hard thresholding and soft thresholding (Zhou et al., 2016).

The hard thresholding function expression is shown in Eq. (4):

(4) \begin{eqnarray*}\overline{{s}_{i,j}}= \left\{ \begin{array}{@{}lr@{}} \displaystyle {s}_{i,j},&\displaystyle \left\vert {s}_{i,j} \right\vert \geq \lambda \\ \displaystyle 0,&\displaystyle \left\vert {s}_{i,j} \right\vert \prec \lambda \end{array} \right. \end{eqnarray*}

where, *s*_*i*,*j*_ is the j-th wavelet coefficient on the i-th scale, and $\overline{{s}_{i,j}}$ is the wavelet coefficient after hard threshold denoising. *λ* is the critical threshold (Badiezadegan & Rose, 2015) .

The soft thresholding function expression is shown in Eq. (5):

(5) \begin{eqnarray*}\overline{{s}_{i,j}}= \left\{ \begin{array}{@{}lr@{}} \displaystyle sgn({s}_{i,j})( \left\vert {s}_{i,j} \right\vert -\lambda ),&\displaystyle \left\vert {s}_{i,j} \right\vert \geq \lambda \\ \displaystyle 0,&\displaystyle \left\vert {s}_{i,j} \right\vert \prec \lambda . \end{array} \right. \end{eqnarray*}

In the above formula, sgn(*) is called Signum function, which is a logic function to judge the sign of its parameters.

When using wavelet threshold denoising, it is necessary to select the appropriate wavelet basis, threshold and threshold function. According to the set parameters, the signal is decomposed into a series of wavelet packet coefficients. After denoising and reconstructing all wavelet packet coefficients according to the threshold function, the denoised signal is obtained.

### Gated recurrent unit neural network

**Figure 1:**
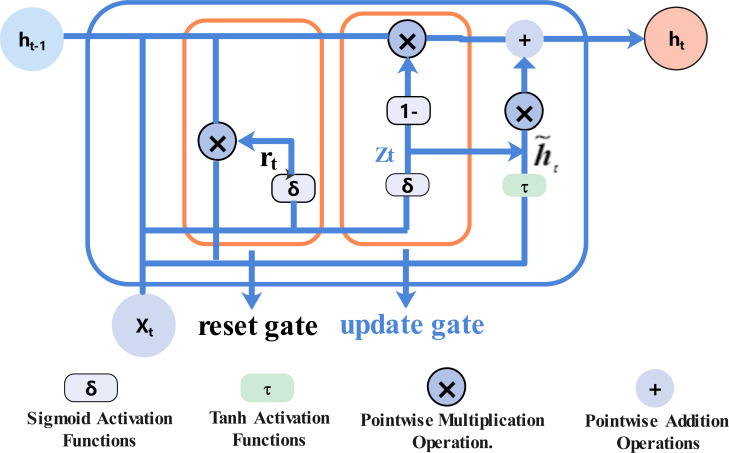
GRU neuron structure diagram.

The gated recurrent unit (GRU) has two gated units, an update gate and a reset gate. Compared with LSTM, the structure is simpler, the number of parameters is fewer, and the model training more easily converges and predicts similar performances. The neuron structure of the GRU neural network is shown in Fig. 1:

The update gate *z*_*t*_ is used to control how much of the previous hidden state enters the current input state, as in Eq. (6).

(6) \begin{eqnarray*}{z}_{t}=\sigma ({W}_{zx}{x}_{t}+{W}_{zh}{h}_{t-1}+{b}_{z}).\end{eqnarray*}

The reset gate *r*_*t*_ reset gate is used to determine the degree of discarding previous information, as in Eq. (7).

(7) \begin{eqnarray*}rt=\sigma ({W}_{rx}{x}_{t}+{W}_{rh}{h}_{t-1}+{b}_{r}).\end{eqnarray*}

In the above formula, *z*_*t*_ is the output of the update gate at time t, *r*_*t*_ is the value of the reset gate at time t, *σ* is the sigmoid activation function, *h*_*t*−1_ is the hidden state at t-1, and *x*_*t*_ is the input vector at the current time. *W*_*rx*_, *W*_*rh*_ and *b*_*r*_ are the corresponding weight matrix and bias vector.

The reset gate output at the current time *r*_*t*_ and the hidden state at the previous time *h*_*t*−1_ are bitwise multiplied. The result of the operation and the input at the current time are used to calculate the candidate hidden state ${\tilde {h}}_{t}$ through the fully connected layer with the activation function tanh, as in eq. (8).

(8) \begin{eqnarray*}{\tilde {h}}_{t}=\tanh \nolimits ({W}_{xh}{x}_{t}+{W}_{hr}({r}_{t}\otimes {h}_{t-1})+{b}_{h}).\end{eqnarray*}

The hidden state *h*_*t*−1_ at the last moment and the current candidate hidden state ${\tilde {h}}_{t}$ perform related operations through the update gate to obtain the current hidden state *h*_*t*_, as in Eq. (9).

(9) \begin{eqnarray*}{h}_{t}={z}_{t}\otimes {\tilde {h}}_{t}+(1-{z}_{t})\otimes {h}_{t-1}.\end{eqnarray*}

The GRU neural network is a time recursive neural network. The gated loop unit can retain relevant information and pass it to the next unit, which fully reflects the long-term historical process of the time series, and is suitable for long-term prediction of the time series.

### Improved whale optimization algorithm

The whale optimization algorithm (Mirjalili & Lewis, 2016) is a swarm intelligence optimization algorithm inspired by whale hunting behavior. The algorithm achieves the goal of global optimization by simulating the three group behaviors of whale searching, encircling and predation. In the whale algorithm, finding a solution to a problem can be understood as the process of whales looking for prey. Whales first search for prey in space and obtain relevant information, and then continue to surround and spiral close to the prey. The behavior of the whale searching for the optimal position can be described by formula (10):

(10) \begin{eqnarray*}{X}_{t+1}={X}_{t}^{\ast }-A \left\vert C{X}_{t}^{\ast }-{X}_{t} \right\vert .\end{eqnarray*}

In the above formula, *X*_*t*_ indicates the position vector of the current iteration, and *t* indicates the current iteration number. A and C represent the coefficient vectors of the convergence factor and the swing factor respectively, and ${X}_{t}^{\ast }$ is the position vector of the best solution obtained thus far. The expressions of the efficient vectors A and C are shown in formula (11) and formula (12) respectively.

(11) \begin{eqnarray*}A=2\vec{a}\vec{r}-\vec{a}\end{eqnarray*}

(12) \begin{eqnarray*}C=2\vec{r}.\end{eqnarray*}

In the above formula, $\vec{r}$ is a random vector with a value range of [0,1], and C is a random number uniformly distributed in (0,2). The initial value of $\vec{a}$ is 2, and linearly decreases to 0 over the course of iterations, as in Eq. (13):

(13) \begin{eqnarray*}\vec{a}=2-2\times \frac{t}{{T}_{\mathrm{max}}} \end{eqnarray*}

where *T*_max_ represents the maximum number of iterations.

However, in the iterative process of the algorithm, the linear change in a cannot effectively reflect the convergence process of the parameters (Ding, Wu & Zhao, 2020; Peng et al., 2021). Therefore, the following nonlinear convergence method is applied, as in Eq. (14):

(14) \begin{eqnarray*}\vec{a}=({a}_{init}-{a}_{final})( \frac{{T}_{\mathrm{max}}-t}{{T}_{\mathrm{max}}} )^{3}.\end{eqnarray*}

In the above formula, *a*_*init*_ and *a*_*final*_ are the initial and final values of parameter $\vec{a}$, respectively, and *T*_max_ is the maximum number of iterations. The improved whale algorithm can ensure that the algorithm accelerates the convergence speed in the early iterations to ensure the global search capability. In the later stage of the iteration, the change in parameters slows down to improve the local search ability of the algorithm (Luan et al., 2019).

The whale algorithm is set so that when $ \left\vert A \right\vert \lt 1$, the whale chooses to swim toward the optimal individual and executes the method of surrounding the prey; when $ \left\vert A \right\vert \geq 1$, the whale cannot obtain effective clues, so it uses a random search for prey. When searching randomly, the positions of other whales are updated according to the positions of the randomly selected whales, to find a more suitable prey, so that the WOA algorithm can perform a global search. As in Eq. (15).

(15) \begin{eqnarray*}{X}_{t+1}={X}_{t}^{r}-A \left\vert C{X}_{t}^{r}-{X}_{t} \right\vert .\end{eqnarray*}

In the above formula, ${X}_{t}^{r}$ is the position vector of the randomly selected whale.

When hunting, humpback whales eject a steam drum to form a bubble net to drive away the prey, and swim to the prey in a spiral motion, so the mathematical formula of hunting behavior is shown in Eq. (16):

(16) \begin{eqnarray*}{X}_{t+1}= \left\vert {X}_{t}^{\ast }-{X}_{t} \right\vert \cdot {e}^{bl}.\cos \nolimits (2\pi l)+{X}_{t}^{\ast }.\end{eqnarray*}

In the above formula, *b* is a logarithmic spiral constant, and *l* is a random number in (−1, 1).

During the hunting process of a school of whales, each whale has a certain possibility to choose to shrink and surround or spiral to approach its prey. The probability p is used to judge the behavior of the whale. When *p* < 0.5, the enveloping contraction method is executed, and formula (10) is used to update the position; when *p* ≥ 0.5, the spiral approach hunting method of formula Eq. (16) is executed.

### Construction of the GRU prediction model based on the improved whale algorithm

**Figure 2:**
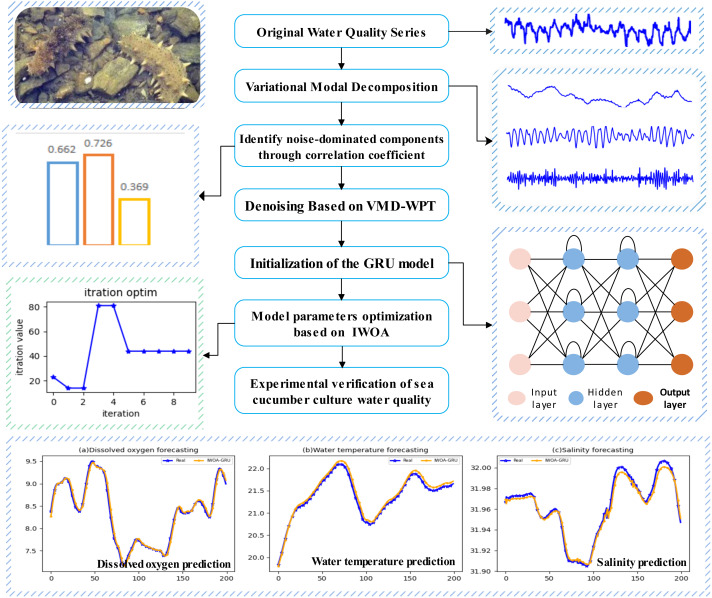
The IWOA-GRU prediction model construction flowchart.

Sea cucumber aquaculture water quality data are easily affected by factors such as temperature, rainfall, man-made operations, and sea cucumber metabolism. It has characteristics such as nonlinearity, a large fluctuation range, and considerable noise, which affect the prediction accuracy. This article uses variational modal decomposition (VMD) to decompose the original time series data, and mines the characteristic information of different time scales in the original signal to achieve data stabilization. By calculating the correlation coefficient between each component and the original data, the noisy component is determined, and the wavelet packet threshold denoising method is used to reduce noise. To improve the prediction performance of the GRU recurrent neural network, the article improves the whale optimization algorithm, applies the improved algorithm to optimize the GRU model parameters, and builds a GRU water quality prediction model based on the improved whale algorithm (IWOA-GRU), and the model construction flowchart as shown in Fig. 2.

## Simulation experiment and result analysis

### Data sources

This article selectes the water quality data of a sea cucumber farming area from a marine ranch in Yantai, Shandong, China for simulation experiments. The Yantai sea area is 26,000 square kilometers, the coastline is 1,038 kilometers long, and it is located near 38 degrees north latitude. It has sufficient sunlight, and the water temperature is between −1.0 and 28 throughout the year; the seawater salinity is between 28 and 32; the pH value is between 7.8 and 8.2. It is the original ecologically good ground for sea cucumbers to inhabit and multiply. The sea cucumber farming area in Yantai is approximately 596,000 mu, accounting for approximately 16.7% of China. Nearly 90% of Yantai sea cucumbers are cultivated by bottom sowing in the sea.

This article used water quality data collected from June 2 to July 1, 2021, for experimental verification. Water quality data were collected every 10 min, including the temperature, salinity and dissolved oxygen of the aquaculture water. After data preprocessing, 4,106 valid data points were obtained. Eighty percent of the sample data were used as the training set to train the prediction model, and the remaining data were used as the test set.

### Evaluation index

This article used mean absolute error (MAE), mean square error (MSE), and the coefficient of determination (*R*^2^) as the evaluation indicators of model prediction performance (Filik & Filik, 2017; Shcherbakov et al., 2013). (1) MAE is the average of the absolute value of the error between the predicted value and the true value. As in Eq. (17):

(17) \begin{eqnarray*}MAE= \frac{1}{N} \sum _{i=1}^{n} \left\vert {y}_{i}-{\hat {y}}_{i} \right\vert .\end{eqnarray*}

(2) MSE refers to the expected value of the square of the difference between the predicted value and the true value; the smaller the value is, the better the accuracy of the prediction model. As in Eq. (18):

(18) \begin{eqnarray*}MSE= \frac{1}{N} \sum _{i=1}^{N}({\hat {y}}_{i}-{y}_{i})^{2}.\end{eqnarray*}

(3) *R*^2^ is generally used to evaluate the degree of linear fit of the prediction model. The closer its value is to 1, the better the prediction performance of the model. As in Eq. (19):

(19) \begin{eqnarray*}{R}^{2}=1- \frac{\sum ({\hat {y}}_{i}-{y}_{i})^{2}}{\sum ({\hat {y}}_{i}-{\bar {y}}_{i})^{2}} .\end{eqnarray*}

In the above three formulas, *y*_*i*_ represents the true value, ${\hat {y}}_{i}$ represents the predicted value, ${\bar {y}}_{i}$ is the average of the true value, and N is the number of samples.

### Data decomposition based on VMD

The VMD decomposition method uses an iterative search for the optimal solution to determine the set of modal components and their respective center frequencies, realizes the effective decomposition of the inherent modal components (IMF) of the nonlinear time series, and obtains a number of different frequency scales and relative stationary subsequence.

The VMD algorithm needs to reasonably set the number of decomposition modes K and the penalty parameter *α*. If the value of k is set too large, the sequence may be overdecomposed, resulting in too many high-frequency modes. If the k value is too small, the sequence will not completely decompose. If the value of *α* is too large, the frequency band information will be lost, otherwise, the information will be redundant. This article used relative entropy to optimize the parameters of VMD and determined the optimal combination of the decomposition level K and the penalty factor *α*.

**Figure 3:**
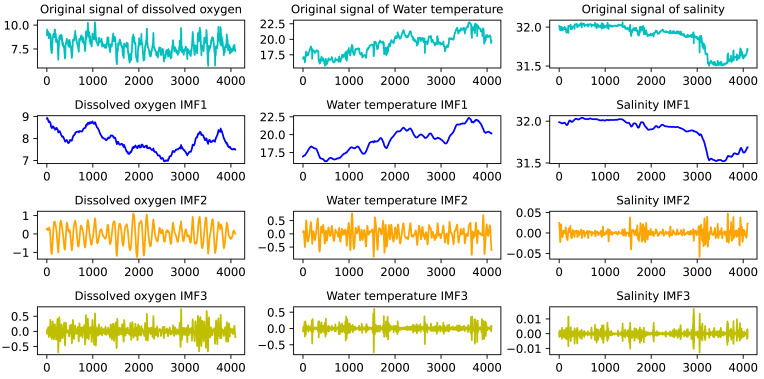
VMD decomposition effect of water quality data.

In this article, by calculating the relative entropy of the intrinsic mode component (IMF) obtained in the iterative decomposition process, the optimal solution of K and *α* corresponding to the minimum relative entropy was obtained. Figure 3 shows the VMD decomposition effect of dissolved oxygen, water temperature, and salinity in sea cucumber farming waters of a marine ranch in Yantai, China. According to the parameter optimization based on relative entropy, the decomposition layer number K was 3, and the value of *α* was 1,350.

The correlation factors between each IMF component obtained by VMD decomposition and the original water quality sequence were calculated, and the IMF components were divided into noise dominant mode and effective information dominant mode according to the correlation analysis. The IMF components whose correlation factor with the original signal was less than 0.5 were processed by wavelet threshold denoising. As shown in Table 1, it is the correlation coefficient between the dissolved oxygen, water temperature, and salinity components and their original sequence.

### Wavelet threshold denoising of noise dominant signals

The wavelet coefficient of the effective signal is greater than the wavelet coefficient of the noise. Therefore, an appropriate threshold is selected, the wavelet coefficient of the effective signal is greater than the threshold, and it is retained. Signals with wavelet coefficients less than the threshold need to be denoised (Wu et al., 2015). The article used the wavelet packet denoising algorithm combining soft and hard thresholds for the abovementioned components whose correlation coefficients after VMD decomposition were less than 0.5. The wavelet base was sym8, and the number of decomposition layers was 3. The threshold function is shown in Eq. (20):

(20) \begin{eqnarray*}\lambda = \frac{median( \left\vert {W}_{1,j} \right\vert )}{0.6745} \sqrt{2\ln \nolimits (N)}.\end{eqnarray*}

**Figure 4:**
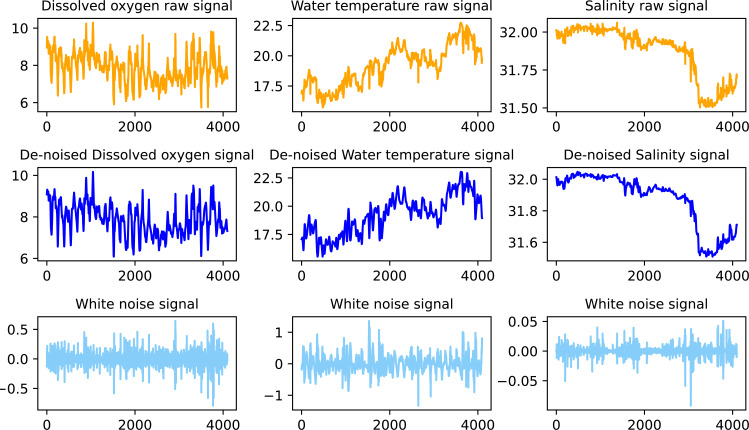
Water quality data denoising effect.

The effect of the original signal after VMD decomposition and wavelet packet threshold denoising is shown in Fig. 4.

**Table 1:** Correlation coefficient between IMF and original data.

Water quality index	IMF1	IMF2	IMF3
Dissolved oxygen	0.662	0.726	0.369
Water temperature	0.983	0.221	0.096
Salinity	0.997	0.092	0.021

### Construction of the water quality prediction model

When using the whale algorithm to train the recurrent neural network, due to the large number of parameters in the recurrent neural network, the difficulty in finding the global optimal solution increases accordingly, the search ability of the algorithm deteriorates, and it easily falls into the local optimal state. In this article, an improved whale algorithm is used to train and optimize the hyperparameters of the GRU recurrent neural network. The specific steps are as follows:

 Step 1: Perform noise reduction processing on the water quality data of sea cucumber farming waters, and determine the training set and test set.

Step 2: Set the number of hidden layers of the GRU cyclic neural network and the number of neurons in each layer, the number of model training iterations, learning rate and other parameters, and construct a parameter vector *w*_*i*_ = {*w*_1,_*w*_2_, …*w*_*n*_}, where n is the number of parameters.

Step 3: Initialize the whale algorithm population size, maximum number of iterations, initial minimum weight and maximum weight and other parameters. Convert the parameter vector in step 2 into the position vector of the improved whale algorithm.

Step 4: Use the mean square error between the output value predicted by the model and the measured value as the fitness function. Calculat the fitness value of each whale and determin the current optimal position vector.

Step 5: Iteratively update the position vector according to the improved optimization strategy. When the maximum number of iterations is met or the error accuracy requirement is met, the optimization algorithm is terminated, and the current optimal parameters are assigned to the GRU prediction model.

step 6: Use the optimized GRU neural network to predict water quality indicators such as dissolved oxygen, water temperature, and salinity, and evaluate the prediction effect.

**Figure 5:**
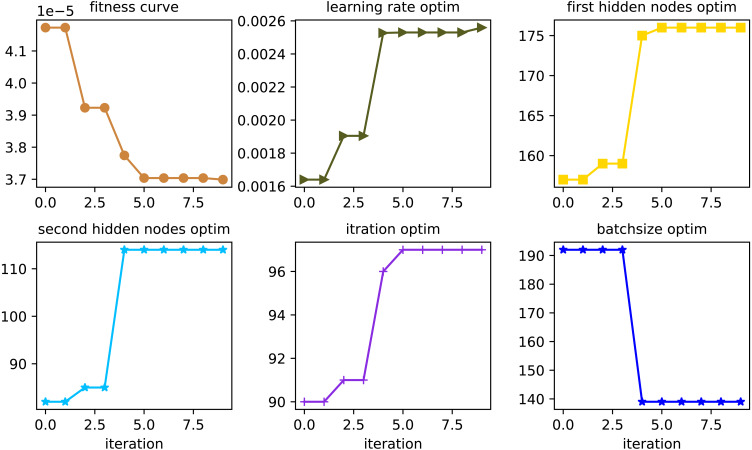
Parameter optimization curve.

Take the denoised water quality data as input samples, and apply the improved whale algorithm in this article to optimize the learning rate, number of iterations, number of hidden layers, and the number of neurons in each layer of the GRU recurrent neural network. By empirical data being selected, and being adjusted through multiple experiments, the parameters of the whale algorithm are set as follows: the number of whales is 50, the maximum number of iterations is 200, and the number of dimensions is six. The position of the whale represents parameters such as the learning rate, the number of iterations, the number of neurons in the first hidden layer, the number of neurons in the second hidden layer, the batchsize, and the timesteps of the GRU model. Taking the dissolved oxygen data prediction as an example, the optimization process of the parameters of the GRU prediction model by the improved whale algorithm is shown in Fig. 5.

### Forecast effect analysis

To verify the prediction performance of the model in this article, support vector regression (SVR), convolutional neural network (CNN), random forest (RF), long short-term memory (LSTM) , and gated recurrent units (GRU) were used to conduct water quality prediction experiments to observe the prediction effects of different models on sea cucumber aquaculture water quality data. To eliminate the contingency of results caused by one experiment, five experiments were carried out on each model, and the average of the results of multiple experiments was taken as the final experimental result. The evaluation indicators of each model are shown in Table 2.

**Table 2:** Forecast model accuracy comparison.

Prediction model	Dissolved oxygen			Water temperature			Salinity		
	MAE	MSE	*R* ^2^	MAE	RMSE	*R* ^2^	MAE	MSE	*R* ^2^
SVR	0.1514	0.0359	0.9235	0.1794	0.0486	0.8963	0.2495	0.0893	0.8297
CNN	0.0668	0.0064	0.9861	0.0530	0.2101	0.9301	0.1965	0.0931	0.8105
RF	0.0798	0.0098	0.9785	0.1373	0.1876	0.8959	0.0859	0.0559	0.9655
RNN	0.0785	0.0165	0.9691	0.1026	0.0217	0.9593	0.0888	0.0184	0.9825
LSTM	0.0449	0.0041	0.9864	0.1010	0.0285	0.9845	0.0961	0.0136	0.9825
GRU	0.0485	0.0059	0.9889	0.0912	0.0191	0.9891	0.0846	0.0169	0.9827
**IWOA-GRU**	**0.0335**	**0.0035**	**0.9983**	**0.0361**	**0.0072**	**0.9954**	**0.0533**	**0.0118**	**0.9947**

**Notes.**

Results for the IWOA-GRU model are shown in bold.

**Figure 6:**
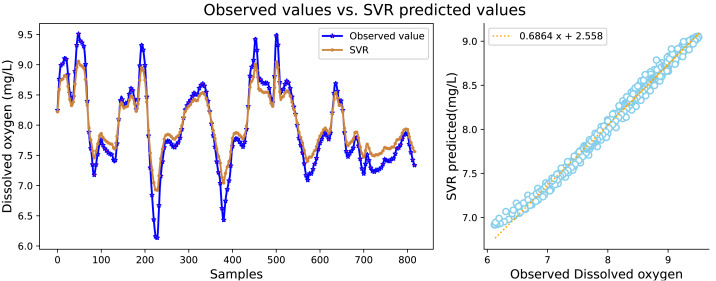
The prediction effect of the SVR model on dissolved oxygen.

**Figure 7:**
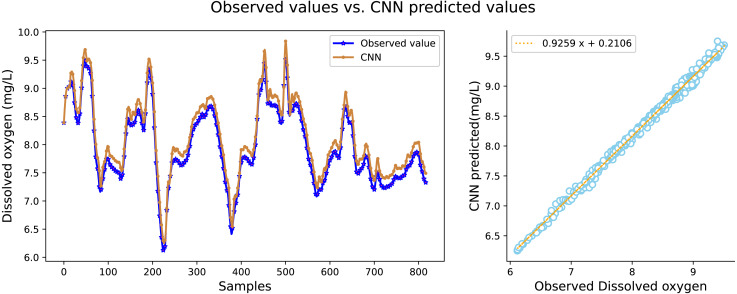
The prediction effect of the CNN model on dissolved oxygen.

**Figure 8:**
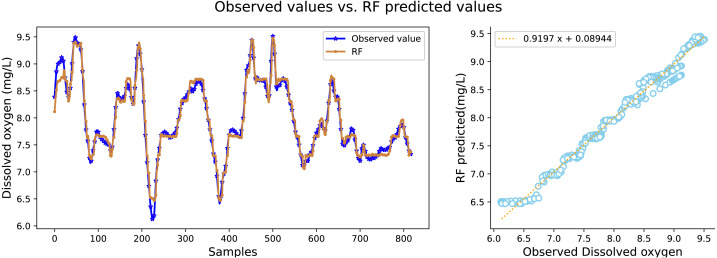
The prediction effect of the RF model on dissolved oxygen.

**Figure 9:**
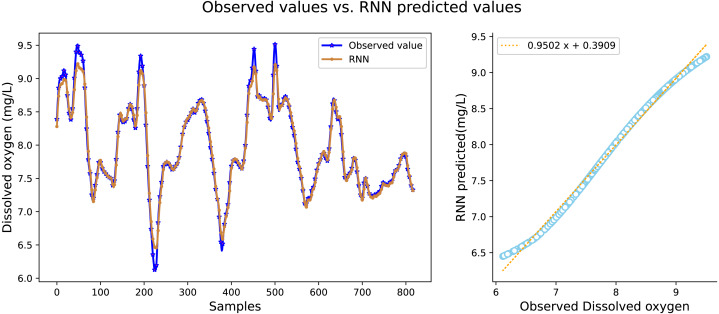
The prediction effect of the RNN model on dissolved oxygen.

**Figure 10:**
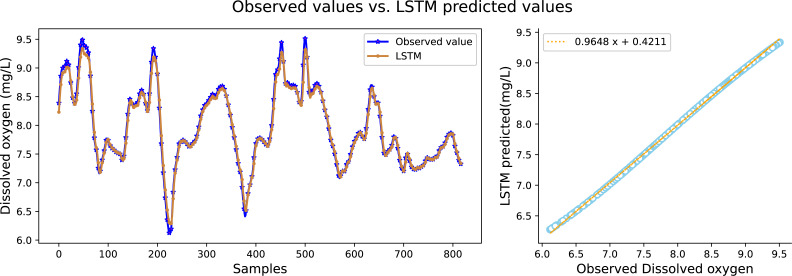
The prediction effect of the LSTM model on dissolved oxygen.

**Figure 11:**
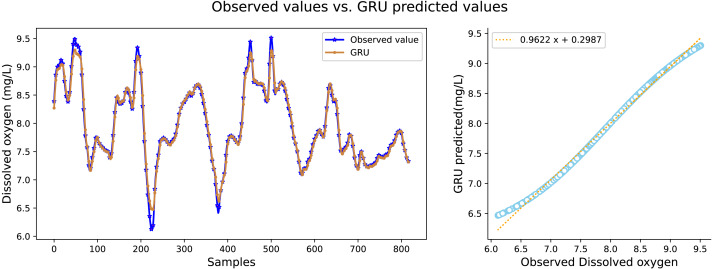
The prediction effect of the GRU model on dissolved oxygen.

**Figure 12:**
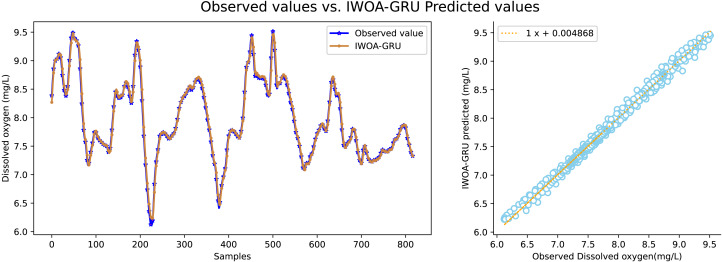
The prediction effect of the IWOA-GRU model on dissolved oxygen.

It can be seen in Table 2 that the water quality prediction model proposed in this article achieves higher prediction accuracy than the other compared models. Among them, the prediction performance of the LSTM and GRU recurrent neural network are equivalent, and the value of *R*^2^ is greater than 98 percent, which is better than prediction models such as RNN, SVR, CNN and RF. The structure of the GRU recurrent neural network is optimized through the improved whale algorithm, which greatly improves its prediction performance. Taking water temperature as an example, MAE decreased by 60.4 percent, MSE decreased by 62.3 percent, and the value of RR increased to more than 99 percent. The experimental results show that the prediction model in this article can predict the water quality of sea cucumber farming with higher precision. The prediction effect of each comparative model on dissolved oxygen is shown in Figs. 6, 7, 8, 9, 10, 11 and 12.

**Figure 13:**
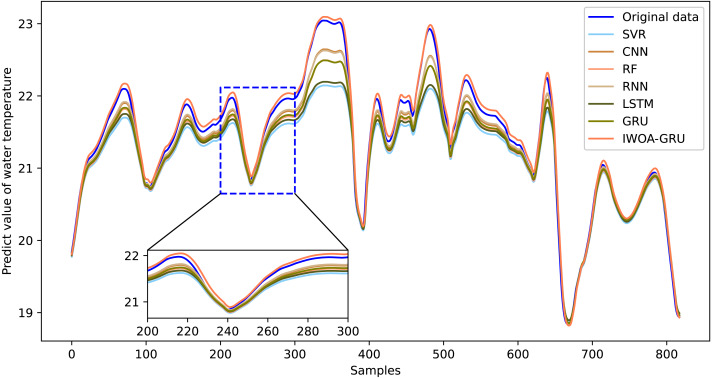
Predictive effect of each comparative model on water temperature.

**Figure 14:**
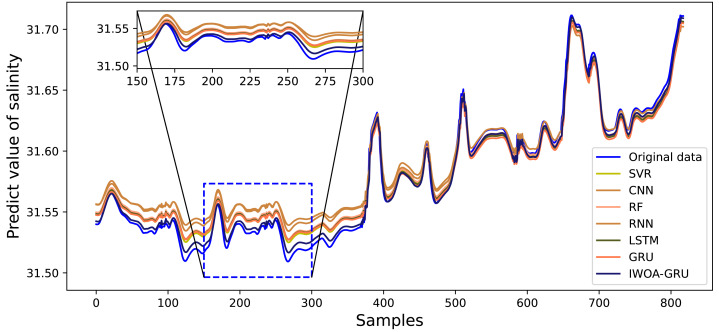
Predictive effect of each comparative model on salinity.

Figures 6, 7, 8, 9, 10, 11 and 12 show that the predicted value on the dissolved oxygen sequence of the IWOA-GRU model in the article is the closest to the true value curve, and the model has the smallest prediction error and the highest degree of linear fit. LSTM neural network and GRU neural network have the characteristics of being suitable for processing time series problems, and simultaneously solving the problem of long-term dependence in the time dimension. The prediction curve fitting effect is better than that of RNN, RF, CNN, and SVR. Through further experimental observation, the prediction effects of each model on water temperature and salinity are shown in Figs. 13 and 14 below.

In Figs. 13 and 14, it can be seen that the water temperature and salinity of sea cucumber farming waters are easily affected by the external environment, there are many data mutations, and the overall stability of the prediction effect is lower than that of dissolved oxygen. The model proposed in the article improves the accuracy and stability of traditional prediction models. The prediction errors are smaller than those of the other compared models, and the overall trend is more consistent with the original data. It also has more accurate predictions for sudden changes and peaks in the data, with the highest degree of fit.

**Figure 15:**
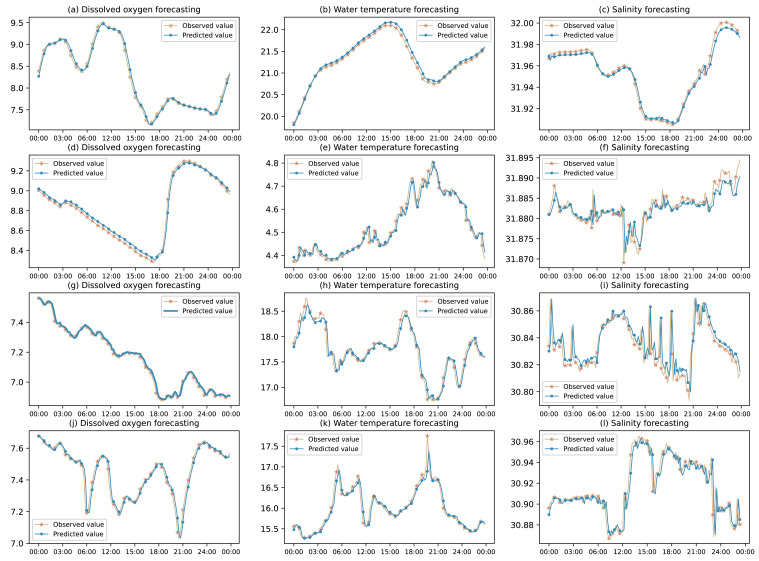
Water quality prediction effect of multiple marine ranches.

To further verify the generalization performance of the prediction model (IWOA-GRU) in this article, the water quality data of the sea cucumber farming area of four marine ranches in the Shandong Peninsula, China are used for further experimental verification. The water quality indicators are dissolved oxygen, water temperature and salinity. The results of the experiment on a certain day are shown in Fig. 15.

As seen in Fig. 15, this model has stable prediction performance when the water quality data change relatively smoothly. When the data undergo large jumps, it can also predict the change trend better and improve the prediction accuracy of the peak value of the sequence. This model has a good fitting effect on the overall change trend of various water quality data and its partial details, and is suitable for predicting the future change trend in sea cucumber aquaculture water quality.

### Conclusions

In this article, the combined noise reduction in VMD decomposition and wavelet threshold can effectively strip the noise in the original data and reduce the influence of noise on prediction accuracy. The GRU neural network solves the long-term dependence of time series data forecasting, and is suitable for short-term or long-term forecasting of water quality time series data. Whether the selection of the learning rate, number of hidden layers, and number of nodes of the GRU prediction model are appropriate will affect its prediction performance. The parameters of the GRU prediction model are optimized through the improved whale algorithm, and the IWOA-GRU water quality prediction model is established by applying the optimal parameter combination, which can greatly improve the prediction accuracy.

The water environment of the sea cucumber farming area is complex, and the model in this article has a good predictive effect on the indicators of water temperature, dissolved oxygen, salinity and other factors that have a greater impact on sea cucumbers growth. In future studies, the mutual influence of water quality indicators will be studied, multivariate predictions will be made, the impact of extreme weather conditions on water quality will be considered, and high-precision water quality prediction models under complex environments will be explored.

## Supplemental Information

10.7717/peerj-cs.1000/supp-1 Supplemental Information 1 Water quality data from sea cucumber breeding area of marine ranch in Shandong Peninsula, China Water quality data were collected from the sea cucumber farming area of marine ranches in Shandong Peninsula, China. Data was collected every ten minutes, and water quality indicators include dissolved oxygen (rjy_con), water temperature (temp) and salinity (salt). The data file is in CSV format.

10.7717/peerj-cs.1000/supp-2 Supplemental Information 2 Python program for iwoa-gru Python program for optimizing the parameters of GRU neural network using improved whale algorithm.

10.7717/peerj-cs.1000/supp-3 Supplemental Information 3 Water quality prediction model Python program for water quality prediction using IWOA-GRU. Taking the collected water quality data of sea cucumber breeding area as the input sample.

## Additional Information and Declarations

### Data Availability

The following information was supplied regarding data availability: The original water quality data is available in the Supplementary File.

## References

[ref-1] Ahmed AN, Othman FB, Afan HA, Ibrahim RK, Fai CM, Hossain MS, Ehteram M, Elshafie A (2019). Machine learning methods for better water quality prediction. Journal of Hydrology.

[ref-2] Aldhyani THH, Al-Yaari M, Alkahtani H, Maashi M (2020). Water quality prediction using artificial intelligence algorithms. Applied Bionics and Biomechanics.

[ref-3] Avila R, Horn B, Moriarty E, Hodson R, Moltchanova E (2018). Evaluating statistical model performance in water quality prediction. Journal of Environmental Management.

[ref-4] Azimi S, Azhdary Moghaddam M, Hashemi Monfared S (2019). Prediction of annual drinking water quality reduction based on Groundwater Resource Index using the artificial neural network and fuzzy clustering. Journal of Contaminant Hydrology.

[ref-5] Badiezadegan S, Rose RC (2015). A wavelet-based thresholding approach to reconstructing unreliable spectrogram components. Speech Communication.

[ref-6] Barzegar R, Asghari Moghaddam A, Adamowski J (2016). Application of wavelet-artificial intelligence hybrid models for water quality prediction: a case study in Aji-Chay River, Iran. Stochastic Environmental Research and Risk Assessment.

[ref-7] Bui DT, Khosravi K, Tiefenbacher J, Nguyen H, Kazakis N (2020). Improving prediction of water quality indices using novel hybrid machine-learning algorithms. Science of the Total Environment.

[ref-8] Cao H, Zhang Z, Zheng Y, Guo H, Zhao R, Shi Y, Chou X (2021). A new joint denoising algorithm for high-G calibration of MEMS accelerometer based on VMD-PE-wavelet threshold. Shock and Vibration.

[ref-9] Ding H, Wu Z, Zhao L (2020). Whale optimization algorithm based on nonlinear convergence factor and chaotic inertial weight. Concurrency and Computation: Practice and Experience.

[ref-10] Dragomiretskiy K, Zosso D (2014). Variational mode decomposition. IEEE Transactions on Signal Processing.

[ref-11] Eze E, Kirby S, Attridge J, Ajmal T (2021). Time series chlorophyll-A concentration data analysis: a novel forecasting model for aquaculture industry. Engineering Proceedings.

[ref-12] Fan S, Hao D, Feng Y, Xia K, Yang W (2021). A hybrid model for air quality prediction based on data decomposition. Information.

[ref-13] Fijani E, Barzegar R, Deo R, Tziritis E, Skordas K (2019). Design and implementation of a hybrid model based on two-layer decomposition method coupled with extreme learning machines to support real-time environmental monitoring of water quality parameters. Science of the Total Environment.

[ref-14] Filik ÜB, Filik T (2017). Wind speed prediction using artificial neural networks based on multiple local measurements in eskisehir. Energy Procedia.

[ref-15] Fu J, Cai F, Guo Y, Liu H, Niu W (2020). An improved VMD-based denoising method for time domain load signal combining wavelet with singular spectrum analysis. Mathematical Problems in Engineering.

[ref-16] Gilles J (2013). Empirical wavelet transform. IEEE Transactions on Signal Processing.

[ref-17] Huan J, Cao W, Qin Y (2018). Prediction of dissolved oxygen in aquaculture based on EEMD and LSSVM optimized by the Bayesian evidence framework. Computers and Electronics in Agriculture.

[ref-18] Huang NE, Shen Z, Long SR, Wu MC, Shih HH, Zheng Q, Yen N-C, Tung CC, Liu HH (1998). The empirical mode decomposition and the Hilbert spectrum for nonlinear and non-stationary time series analysis. Proceedings of the Royal Society of London. Series A: Mathematical, Physical and Engineering Sciences.

[ref-19] Kennedy J, Eberhart R (1995). Particle swarm optimization.

[ref-20] Lei Z, Su W, Hu Q (2019). Multimode decomposition and wavelet threshold denoising of mold level based on mutual information entropy. Entropy.

[ref-21] Liu S, Xu L, Li D (2016). Multi-scale prediction of water temperature using empirical mode decomposition with back-propagation neural networks. Computers & Electrical Engineering.

[ref-22] Lu H, Ma X (2020). Hybrid decision tree-based machine learning models for short-term water quality prediction. Chemosphere.

[ref-23] Luan F, Cai Z, Wu S, Jiang T, Li F, Yang J (2019). Improved whale algorithm for solving the flexible job shop scheduling problem. Mathematics.

[ref-24] Mirjalili S, Lewis A (2016). The whale optimization algorithm. Advances in Engineering Software.

[ref-25] Mirjalili S, Mirjalili SM, Lewis A (2014). Grey wolf optimizer. Advances in Engineering Software.

[ref-26] Nie Z, Wang K, Zhao M (2018). Application of wavelet and EEMD joint denoising in nonlinear ultrasonic testing of concrete. Advances in Materials Science and Engineering.

[ref-27] Niu H, Xu K, Wang W (2020). A hybrid stock price index forecasting model based on variational mode decomposition and LSTM network. Applied Intelligence.

[ref-28] Noori N, Kalin L, Isik S (2020). Water quality prediction using SWAT-ANN coupled approach. Journal of Hydrology.

[ref-29] Peng H, Wen W-S, Tseng M-L, Li L-L (2021). A cloud load forecasting model with nonlinear changes using whale optimization algorithm hybrid strategy. Soft Computing.

[ref-30] Rehman Nu, Aftab H (2019). Multivariate variational mode decomposition. IEEE Transactions on Signal Processing.

[ref-31] Ren Q, Wang X, Li W, Wei Y, An D (2020). Research of dissolved oxygen prediction in recirculating aquaculture systems based on deep belief network. Aquacultural Engineering.

[ref-32] Ren Y, Suganthan PN, Srikanth N (2015). A comparative study of empirical mode decomposition-based short-term wind speed forecasting methods. IEEE Transactions on Sustainable Energy.

[ref-33] Shcherbakov MV, Brebels A, Shcherbakova NL, Tyukov AP (2013). A survey of forecast error measures. World Applied Sciences Journal.

[ref-34] Shi P, Li G, Yuan Y, Huang G, Kuang L (2019). Prediction of dissolved oxygen content in aquaculture using clustering-based softplus extreme learning machine. Computers and Electronics in Agriculture.

[ref-35] Wu Z, Huang NE (2009). Ensemble empirical mode decomposition: a noise-assisted data analysis method. Advances in Adaptive Data Analysis.

[ref-36] Wu Z-G, He C, Xing J, Li J, Yang Q, Wang R (2015). A new wavelet threshold determination method considering interscale correlation in signal denoising. Mathematical Problems in Engineering.

[ref-37] Xu L, Liu S (2013). Study of short-term water quality prediction model based on wavelet neural network. Mathematical and Computer Modelling.

[ref-38] Xue J, Shen B (2020). A novel swarm intelligence optimization approach: sparrow search algorithm. Systems Science & Control Engineering.

[ref-39] Yan J, Liu J, Yu Y, Xu H (2021). Water quality prediction in the luan river based on 1-DRCNN and bigru hybrid neural network model. Water.

[ref-40] Yeh J-R, Shieh J-S, Huang NE (2010). Complementary ensemble empirical mode decomposition: a novel noise enhanced data analysis method. Advances in Adaptive Data Analysis.

[ref-41] Zhang W, Qu Z, Zhang K, Mao W., Ma Y, Fan X (2017). A combined model based on CEEMDAN and modified flower pollination algorithm for wind speed forecasting. Energy Conversion and Management.

[ref-42] Zhou H, Jing-yi L, Hong L, Dong Y, Yan-sheng Z (2016). A new wavelet threshold function and denoising application. Mathematical Problems in Engineering.

[ref-43] Zhu Y, Wang Q, Wang Y, Yuan S., Tang S, Zheng Z (2021). A novel extraction method for useful component of vibration signal combining variational mode decomposition and relative entropy. AIP Advances.

[ref-44] Zou Q, Xiong Q, Li Q, Yi H, Yi H, Yu Y, Wu C (2020). A water quality prediction method based on the multi-time scale bidirectional long short-term memory network. Environmental Science and Pollution Research.

